# Associations of actigraphy derived rest activity patterns and circadian phase with clinical symptoms and polysomnographic parameters in chronic insomnia disorders

**DOI:** 10.1038/s41598-022-08899-2

**Published:** 2022-03-22

**Authors:** Hyun Woong Roh, Su Jung Choi, Hyunjin Jo, Dongyeop Kim, Jung-gu Choi, Sang Joon Son, Eun Yeon Joo

**Affiliations:** 1 Department of Psychiatry, Ajou University School of Medicine, Suwon, Republic of Korea; 2 Department of Brain Science, Ajou University School of Medicine, Suwon, Republic of Korea; 3 Graduate School of Clinical Nursing Science, Sungkyunkwan University, Seoul, Republic of Korea; 4 Department of Neurology, Neuroscience Center, Samsung Biomedical Research Institute, Samsung Medical Center, Sungkyunkwan University School of Medicine, 81 Irwon-ro, Gangnam-gu, Seoul, 06351 Republic of Korea; 5 Department of Neurology, Seoul Hospital, Ewha Womans University College of Medicine, Seoul, Republic of Korea; 6 Yonsei Graduate Program in Cognitive Science, Yonsei University, Seoul, Republic of Korea

**Keywords:** Neuroscience, Neurology

## Abstract

We explored the associations of actigraphy-derived rest-activity patterns and circadian phase parameters with clinical symptoms and level 1 polysomnography (PSG) results in patients with chronic insomnia to evaluate the clinical implications of actigraphy-derived parameters for PSG interpretation. Seventy-five participants underwent actigraphy assessments and level 1 PSG. Exploratory correlation analyses between parameters derived from actigraphy, PSG, and clinical assessments were performed. First, participants were classified into two groups based on rest-activity pattern variables; group differences were investigated following covariate adjustment. Participants with poorer rest-activity patterns on actigraphy (low inter-day stability and high intra-daily variability) exhibited higher insomnia severity index scores than participants with better rest-activity patterns. No between-group differences in PSG parameters were observed. Second, participants were classified into two groups based on circadian phase variables. Late-phase participants (least active 5-h and most active 10-h onset times) exhibited higher insomnia severity scores, longer sleep and rapid eye movement latency, and lower apnea–hypopnea index than early-phase participants. These associations remained significant even after adjusting for potential covariates. Some actigraphy-derived rest-activity patterns and circadian phase parameters were significantly associated with clinical symptoms and PSG results, suggesting their possible adjunctive role in deriving plans for PSG lights-off time and assessing the possible insomnia pathophysiology.

## Introduction

The circadian clock is a system that enables organisms to adapt to external changes in the environment over a 24-h period and is a major determinant of rest-activity patterns, sleepiness, and alertness in humans^1^. Growing evidence suggests that homeostatic sleep drive (Process S) and the circadian pacemaker (Process C) reciprocally interact to regulate the sleep–wake cycle^2^. For example, animals lacking core clock machinery, such as *Bmal1* or *Clock* genes, exhibit impairments in the circadian pacemaker and changes in sleep and quality, such as altered EEG delta power^3–5^. Alterations in rest-activity patterns and circadian phase have also been reported in patients with bipolar disorder, Parkinson’s disease, and Alzheimer's disease^6–10^. Indeed, circadian rhythm disruptions and sleep difficulties are frequently observed in patients with these conditions, suggesting pathophysiological roles for sleep and circadian alterations in disease initiation and progression^11^. We thus consider that although circadian rhythm and sleep are two different aspects of sleep–wake cycle biology, these two factors are not independent and could be correlated with each other. Nevertheless, rest-activity pattern changes, circadian phase alterations, and their possible associations with subjective and objective sleep characteristics in patients with insomnia remain poorly characterized. In 2014, Natale et al. assessed the rest-activity patterns in 151 patients with insomnia and 342 normal sleepers^12^. In this study, there were no significant differences in the rest-activity pattern parameters’ inter-day stability (IS) and intra-daily variability (IV) between patients with insomnia and normal sleepers. However, the absence of these differences does not eliminate the possibility of associations between rest-activity patterns and sleep characteristics in patients with insomnia. For example, the subset of patients with insomnia could have combined rest-activity pattern disruption (low IS and high IV), and this combined difficulty can exacerbate patients’ subjective suffering from insomnia. In a similar way, the circadian phase of patients with insomnia could also have a possible impact on sleep disturbance in each individual.

Polysomnography (PSG) is considered the gold standard for objectively evaluating sleep quality and quantity^13^. PSG provides an accurate measure of wake and sleep times, as well as respiratory function, muscle activity, heart physiology, and sleep stages. However, level 1 PSG is typically performed for one night in a specialized setting, regardless of patients’ lifestyle, such as sleep environment, which may contribute to poor ecological validity^14^. Sleep diaries and/or questionnaires are commonly used to identify patients’ lifestyle and circadian phase^15,16^. Actigraphy has emerged as a major assessment tool in sleep research and sleep medicine over the last two decades^17,18^. In this context, patients’ rest-activity patterns and circadian phase derived from several days of actigraphy assessment may be helpful for performing and/or interpreting level 1 PSG in clinical practice. Several studies have compared PSG-derived sleep parameters with actigraphy-derived parameters such as total sleep time and wakefulness after sleep onset^19–21^. However, to the best of our knowledge, no study to date has evaluated the clinical significance of actigraphy-derived rest-activity patterns and circadian phase parameters for the preparation and interpretation of level 1 PSG.

In this study, we investigated the possible associations of actigraphy-derived rest-activity patterns and circadian phase parameters with clinical symptoms and PSG results in 75 patients with insomnia in order to assess the possible associations of the rest-activity pattern or circadian phase deviation with clinical symptoms or PSG results. In addition, we also tried to assess the clinical implications of actigraphy-derived parameters for the preparation and interpretation of PSG in patients with insomnia.

## Results

### Demographic characteristics, rest-activity patterns, circadian phase, and PSG results

Demographic characteristics, rest-activity patterns, and circadian phase according to actigraphy, PSG results, and clinical symptom scores of the study participants are listed in Table 1. In brief, the median (interquartile range, IQR) age was 58 (51–65) years and body mass index (BMI) was 22.2 (20.4–24.2). Of the 75 participants, 59 (78.7%) were female. Of the participants, 66 (88.0%) wore the actigraphy device over the entire 7 days, and nine (12.0%) reported 1–2 missing days during the recording period. Rest-activity pattern variables (IS and IV) and circadian phase variables (least active 5-h (L5) and most active 10-h (M10) onset times) were extracted from the actigraphy measurements. The mean and median values of these parameters did not deviate substantially from our previous findings in elderly individuals with cognitive impairments and other studies assessing similar parameters^7,8,22^. PSG results indicated that total sleep time and wake after sleep onset were approximately 361.9 min and 14.4%, respectively.

**Table 1:** Demographic characteristics, rest-activity patterns, circadian phase, and level 1 PSG parameters of study participants.

Variables^a^	All participants (n = 75)
Age, year	58 (51–65)
Female, No. (%)	59 (78.7)
Height, cm	158.0 (154.0–163.0)
Weight, kg	56.0 (51.0–62.0)
Body mass index	22.2 (20.4–24.2)
**Actigraphy recording days, No. (%)**	
5 days	3 (4.0)
6 days	6 (8.0)
7 days	66 (88.0)
**Rest-activity patterns and circadian phase on actigraphy**	
IS, day to day consistency	0.56 (0.46–0.64)
IV, fragmentation of activity	0.81 (0.20)
L5 onset time, rest phase, time	24.5 (1.0)
M10 onset time, active phase, time	9.5 (2.1)
**Sleep parameters on actigraphy**	
Total sleep time, min	358.0 (60.9)
Sleep latency, min	4.0 (1.8–9.2)
Sleep efficiency, %	85.5 (78.8–89.5)
Wakefulness after sleep onset, %	11.1 (8.2–15.7)
**Polysomnography (level 1) results**	
Total sleep time, min	361.9 (61.6)
Sleep latency, min	10.5 (6.5–28.5)
REM latency, min	97.5 (74.0–154.0)
Sleep efficiency, %	82.0 (74.1–88.2)
Wakefulness after sleep onset, %	14.4 (8.0–23.2)
Sleep stage N1, %	14.4 (9.7–18.9)
Sleep stage N2, %	61.4 (10.0)
Sleep stage N3, %	0.8 (0.0–3.6)
Sleep stage REM, %	18.5 (6.0)
Apnea Hypopnea Index	7.7 (2.7–19.4)
Clinical symptom score^b^	
Insomnia severity index	18.3 (4.7)
Epworth sleepiness scale	4.5 (2.0–7.0)

*IQR* interquartile range, *IS* inter-day stability, *IV* intra-daily stability, *L5 onset time* least active 5-h onset time, *M10 onset time* most active 10-h onset time, *SD* standard deviation.

^a^Values are presented as mean (SD) for normally distributed continuous variables, median (IQR) for non-normally distributed continuous variables, and number (%) for categorical variables.

^b^Five participants did not complete the insomnia severity index (n = 70), and one participant did not complete the Epworth sleepiness scale (n = 74).

### Exploratory correlation analyses of rest-activity patterns, circadian phase, PSG parameters, and clinical symptom scores

To explore the associations among rest-activity patterns, circadian phase, PSG parameters, and clinical symptom scores, Pearson or Spearman correlation analyses were performed based on the distribution of variables (Table 2). As expected, a negative association was noted between actigraphy-derived IS and IV (P < 0.001), and a positive association was observed between L5 and M10 onset times (P < 0.001). In addition, actigraphy-derived L5 onset time, indicative of the rest phase, was negatively associated with apnea–hypopnea index (AHI) (P = 0.05) and positively associated with rapid eye movement (REM) latency (P = 0.006) and the Insomnia Severity Index (ISI) (P = 0.03). M10 onset time, indicative of the active phase, was positively associated with REM latency (P = 0.04). These results suggest that the actigraphy-derived circadian phase might be associated with insomnia severity and some PSG parameters such as AHI and REM latency in patients with insomnia disorder.

**Table 2:**
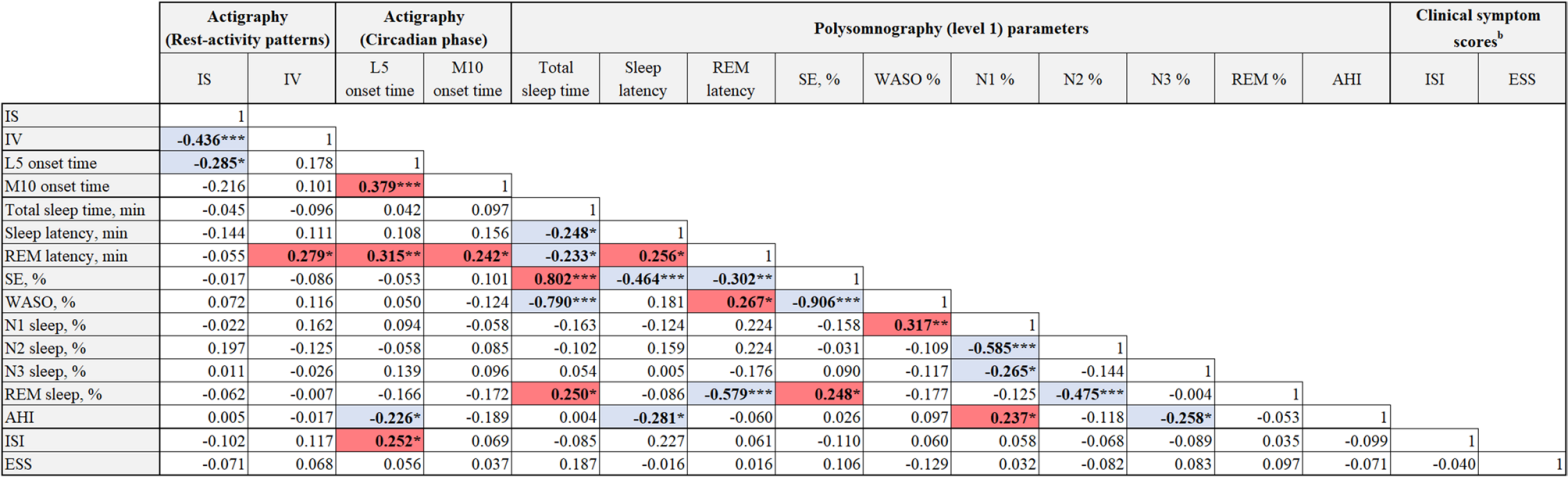
Exploratory correlation analyses among rest-activity patterns, circadian phase, level 1 PSG parameters, and clinical symptom scores^a^.

*AHI* apnea–hypopnea index, *ESS* Epworth sleepiness scale, *IS* inter-day stability, *ISI* insomnia severity index, *IV* intra-daily stability, *L5 onset time* least active 5-h onset time, *M10 onset time* most active 10-h onset time, *REM* rapid eye movement, *WASO* wakefulness after sleep onset.

^a^Pearson correlation test was performed between two normally distributed continuous variables. Otherwise, Spearman rank correlation test was performed. Red and blue boxes indicate significant positive and negative associations, respectively.

^b^Five participants did not complete the insomnia severity index (n = 70), and one participant did not complete the Epworth sleepiness scale (n = 74).

### Data-driven group classification based on actigraphy-derived rest-activity patterns and circadian phase

Hierarchical clustering analyses were performed for the actigraphy data-driven group classification of the participants for the following reasons. First, rest-activity patterns and circadian phase alterations may only occur in a subset of patients with chronic insomnia in sleep clinics. Second, the cut-off values for actigraphy-derived rest-activity patterns or circadian phase parameters have not been suggested to date. Third, using a data-driven clustering approach, two rest-activity pattern variables, IS and IV, or two circadian phase variables, L5 and M10, can be simultaneously considered.

**Figure 1:**
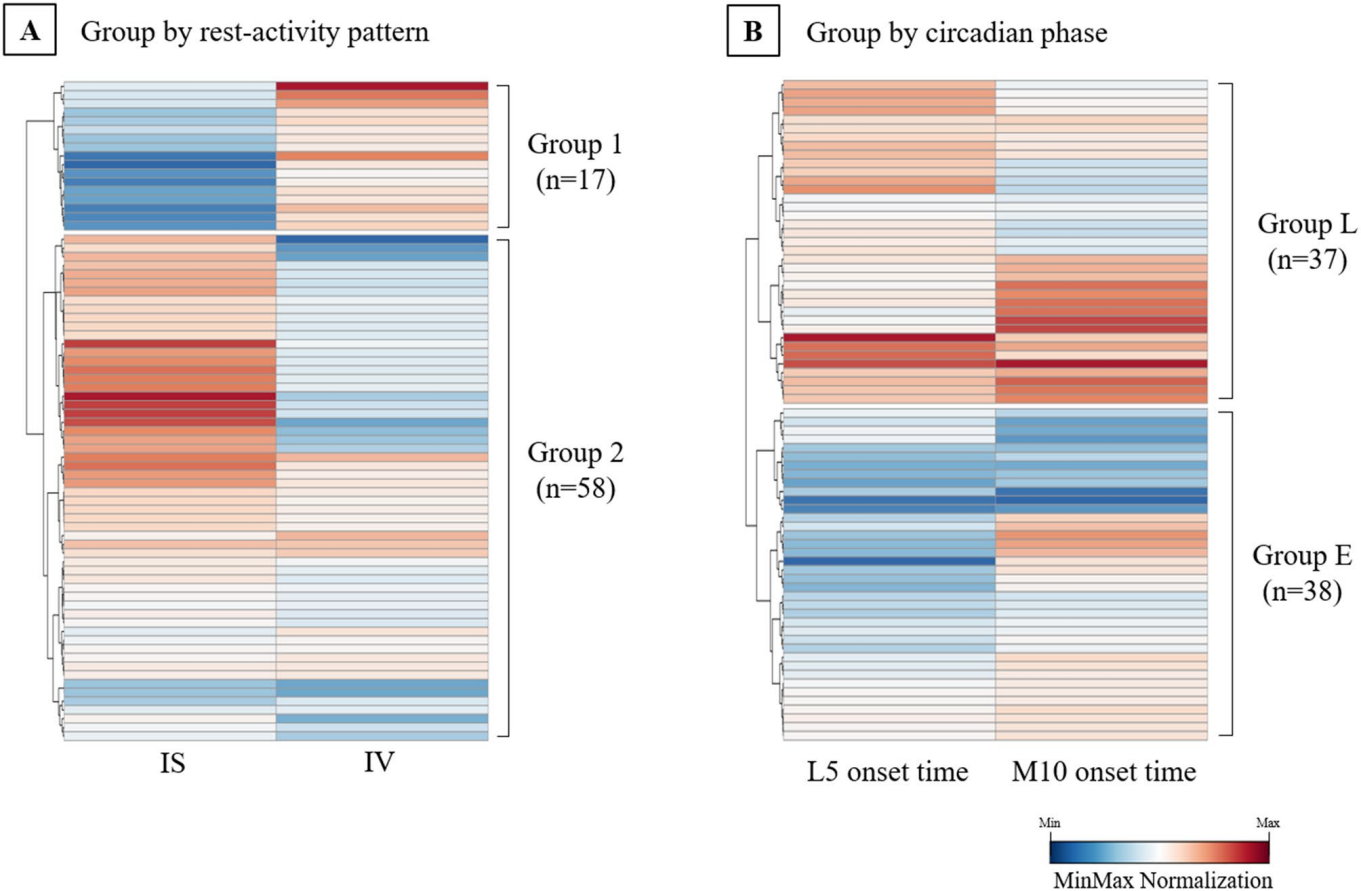
Data-driven group classification of participants based on actigraphy-derived rest-activity patterns and circadian phase. Hierarchical clustering analysis was performed for the data-driven group classification. (**A**) Groupclassification according to the rest-activity patterns. (**B**) Group classification according to the circadian phase. In (**B**), the L5 onset time and M10 onset time were converted to the numeric values. For example, the L5 onset time of 11:30 p.m. was treated as 23.5, and the L5 onset time of 01:15 a.m. was treated as 25.25. We performed Min–Max normalization in each variable for visualization. *IS* inter-daily stability, *IV* intra-daily variability, *L5 onset time* least active 5-h onset time, *M10 onset time* most active 10-h onset time.

First, the study participants were classified into two groups based on rest-activity pattern variables, IS and IV (Fig. 1A). Group classification according to the rest-activity patterns clustered 17 participants with low IS and high IV (Group 1), and 58 participants with high IS and low IV (Group 2). Second, study participants were classified into two groups based on circadian phase variables L5 and M10 onset time (Fig. 1B). Group classification according to circadian phase clustered 37 late-phase participants (group L) and 38 early-phase participants (group E).

### Differences in PSG parameters and clinical symptom scores according to rest-activity pattern variables

**Figure 2:**
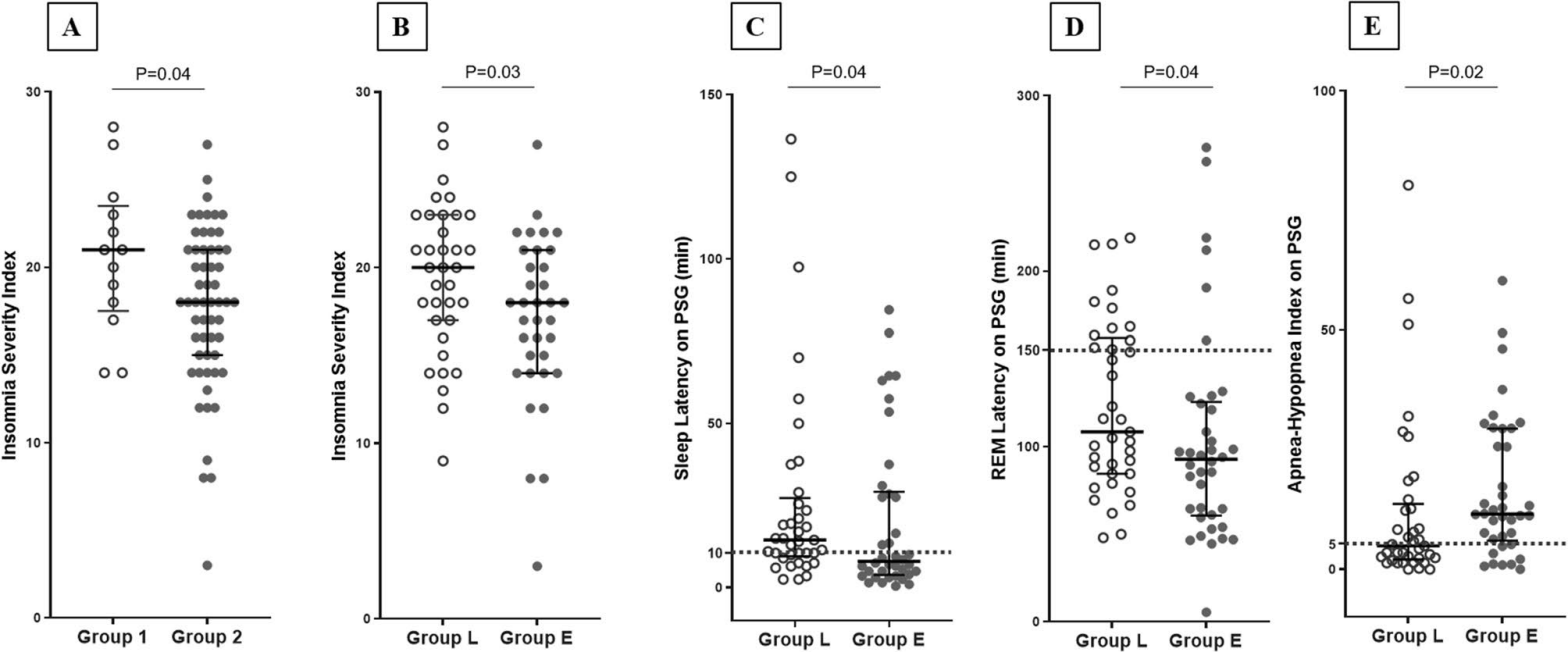
Differences in clinical symptom scores and PSG parameters between groups according to rest-activity patterns and circadian phase (**A–E**). Group 1 (low IS and high IV), Group 2 (high IS and low IV), Group L (late phase), and Group E (early phase). Student's t-test was performed to analyze normally distributed continuous variables (ISI), and the Mann–Whitney U test was conducted to analyze non-normally distributed continuous variables (sleep latency and AHI). Five participants did not complete the insomnia severity index (n = 70). Circles indicate patients. Bars in the middle indicate medians, while error bars indicate interquartile range. Dotted lines in (**C**), (**D**), and (**E**) indicate the conventional cut-off points for each PSG parameter. *AHI* apnea–hypopnea index, *IS* inter-daily stability, *IV* intra-daily variability, *L5 onset time* least active 5-h onset time, *M10 onset time* most active 10-h onset time.

To investigate between-group differences and their clinical implications according to rest-activity pattern variables (IS and IV), PSG parameters and clinical symptom scores were compared between groups. Participants with poorer rest-activity patterns (Group 1, low IS, and high IV) had significantly higher ISI scores in the clinical assessment (P = 0.04; Fig. 2A). This difference remained significant after adjusting for age, sex, BMI, and lights-off time by applying analysis of covariance (ANCOVA) (estimated marginal means [standard error], Group 1: 20.8 [1.3] vs Group 2: 17.7 [0.6]; P = 0.04). No significant between-group differences were noted in other PSG parameters or Epworth Sleepiness Scale (ESS) scores.

### Differences in PSG parameters and clinical symptom scores according to circadian phase variables

To investigate between-group differences and their clinical implications according to circadian phase variables (L5 and M10 onset times), PSG parameters and clinical symptom scores were compared between groups. Late-phase participants (Group L) had significantly higher ISI scores in the clinical assessment (P = 0.03; Fig. 2B). This difference remained significant after adjusting for age, sex, BMI, and lights-off time by applying ANCOVA (estimated marginal means [standard error], Group L: 19.6 [0.8] vs Group E: 17.0 [0.8]; P = 0.03). In addition, late-phase participants (Group L) exhibited significantly longer sleep latency (P = 0.04; Fig. 2C), REM latency (P = 0.04; Fig. 2D), and lower AHI (P = 0.02; Fig. 2E) on PSG. Considering the skewed distribution of the parameters and clinical implications of sleep latency, REM latency, and AHI, logistic regression analysis was performed using conventional cut-off scores after adjusting for age, sex, BMI, and lights-off time. Conventional cut-off scores were defined as 10 min for sleep latency, 150 min for REM latency, and 5 for AHI^23–26^. Compared to early-phase participants (Group E), late-phase participants (Group L) had adjusted odds ratios (ORs) of 5.8 (95% confidence interval [CI] 1.8–18.2, P = 0.003) for long sleep latency, 4.0 (95% CI 1.2–13.6, P = 0.04) for long REM latency, and 0.26 (95% CI 0.07–0.94, P = 0.04) for abnormal AHI.

## Discussion

The present study explored the associations of actigraphy-derived rest-activity patterns and circadian phase parameters with clinical symptoms and level 1 PSG results in 75 patients with chronic insomnia at a sleep clinic. Exploratory correlation analyses revealed significant associations of actigraphy-derived circadian phase parameters (L5 and M10 onset times) with insomnia severity and level 1 PSG parameters such as AHI or REM latency. The 75 subjects were further categorized into two groups based on rest-activity patterns or circadian phase using data-driven hierarchical clustering analysis. The analysis revealed that participants with poorer rest-activity patterns (low IS and high IV) exhibited higher insomnia severity scores compared to participants with better rest-activity patterns (high IS and low IV). In addition, late-phase participants (late L5 and M10 onset times) exhibited higher insomnia severity scores, longer sleep latency, longer REM latency, and lower AHI compared to early-phase participants (early L5 and M10 onset times). These associations remained significant even after adjusting for potential covariates including age, sex, BMI, and lights-off time.

There are several implications that arise from the present study. We did not identify strong relationships between actigraphy-derived rest-activity pattern parameters (IS and IV) and level 1 PSG results in patients with chronic insomnia. IS and IV are the most well-described rest-activity pattern parameters that measure the similarity of one 24-h period to the next and the strength of consolidation of the rest-activity rhythm within a 24-h period, respectively. Several studies have reported possible associations of IS and IV with the differential diagnosis, severity, and pathophysiology of diseases such as Alzheimer’s disease, Parkinson’s disease, bipolar disorder, and posttraumatic stress disorder^8,9^. Changes in rest-activity patterns and circadian rhythm disruptions have been suggested to play pathophysiological roles in these conditions^11,27^ as well as in the initiation and progression of insomnia disorder^13,27–29^. In the present study, we only identified a weak nonparametric positive association between IV and REM latency. However, following categorization of participants into two groups based on IS and IV (Group 1 and Group 2), no significant difference in REM latency was observed between the two groups (data not shown, P = 0.07, Mann–Whitney U test). In addition, other major level 1 PSG parameters such as total sleep time (TST), SL, wakefulness after sleep onset (WASO), N1/N2/N3/REM proportion, and AHI were not significantly associated with IS or IV. These findings are not entirely unexpected given the multifactorial pathophysiology of insomnia disorder^30,31^. Although there is currently no consensus on the proportion of patients with insomnia with circadian rhythm disruptions, the normal distribution of IS and IV parameters in our study suggests that only a subset of patients with chronic insomnia have abnormal rest-activity patterns and circadian rhythms. Another possibility is that actigraphy-derived rest-activity pattern parameters (IS and IV) are factors independent of insomnia severity, regardless of PSG parameters. Indeed, there is a discrepancy between objective measurements of sleep and subjective insomnia^32^. This common misperception of sleep in patients with insomnia is problematic because patients perceive that they are getting insufficient sleep. Therefore, patients with insomnia that have sleep misperception might tend to spend more time on sleep than necessary, even during the daytime, which could result in inappropriate and irregular rest-activity patterns.

Participants with late circadian phase on actigraphy (Group L) presented with more complaints regarding insomnia symptoms and longer sleep and REM latency in PSG. Although this association may seem obvious, our findings have important clinical implications for the performance and interpretation of PSG in sleep clinics. The ecological validity of PSG has been questioned due to the disparity between the specialized environment required for level 1 PSG and patients’ home setting. For example, longer sleep or REM latency on PSG is typically considered to indicate difficulties with sleep initiation in the daily lives of patients. However, it could also be due to delayed circadian phase or discomfort induced by the numerous devices required for level 1 PSG on the day of assessment. Differentiating these factors in clinical practice is critical, but may be challenging due to limited information about the patients’ circadian phase or degree of discomfort in PSG. In the present study, actigraphy-derived circadian phase was calculated, revealing that patients with delayed circadian phase had more abnormal sleep and REM latency based on conventional cut-off scores. Thus, our findings imply a possible adjunctive role of actigraphy-derived circadian phase information for the performance and interpretation of PSG. For instance, patients with early circadian phase on actigraphy but abnormal sleep and REM latency on PSG may have more severe sleep initiation difficulties in daily life or feel more uncomfortable during PSG. In contrast, for patients with delayed circadian phase on actigraphy, our findings suggest that sleep physicians should assess patients’ PSG lights-off time based on patients’ routine sleep schedule reports and actigraphy-measured circadian phase before PSG. Theoretically, PSG lights-off time should be consistent with patients’ routine sleep schedule. However, in clinical practice, there may be discrepancies between PSG lights-off time and patients’ routine sleep schedule or circadian phase due to limited and imprecise information reported by patients. We conjecture that actigraphy-measured circadian phase may provide additional adjunctive information to sleep physicians for deriving plans for PSG lights-off time. In addition, sleep difficulties of patients with delayed circadian phase on actigraphy and abnormal sleep and REM latency on PSG may be due to initiation of sleep that is too early for patients’ circadian phase. Indeed, Flynn-Evans et al. analyzed dim light melatonin onset with sleep logs in 79 patients with insomnia and reported that a substantial proportion (10–22%) of patients with insomnia initiated sleep too early for their circadian phase^33^. For these cases, reassessing circadian rest-activity phase in daily life and applying chronotherapy, melatonin, and/or timed light exposure are potential options for addressing sleep difficulties^34–36^.

Finally, participants with early circadian phase on actigraphy (Group E) exhibited a slightly higher AHI on PSG. This group difference could be caused by confounding factors. Indeed, participants in Group E were older than subjects in Group L (mean age of 60.6 and 53.7 years in Groups E and L, respectively), but no differences in sex or BMI were noted. However, after adjusting for possible covariates including age, subjects in Group E were still significantly more prone to have abnormal AHI on PSG. One large epidemiologic sleep study reported increased AHI values in morning- and evening-type individuals after stratifying the sample by BMI and age^37^. The association between higher AHI values and early circadian phase observed in our study may reflect the protective role of a normal circadian phase in obstructive sleep apnea severity.

This study has several limitations. Considering that all participants had insomnia disorder, associations of rest-activity patterns and circadian phase parameters with clinical symptoms and PSG results concerning control participants may have been missed in our study. We thus could not assess whether the obtained results in our study are specific for patients with insomnia or not. Future studies that include control participants with clinical evaluation and PSG results are warranted. In addition, this study was conducted using a relatively small sample size. It should also be noted that participants in our study were predominantly middle-aged to older adults. These points may limit the generalizability of our results at a population level. Furthermore, our study was cross-sectional; hence, causal relationships could not be conclusively demonstrated.

In conclusion, this study investigated the associations of actigraphy-derived rest-activity patterns and circadian phase with level 1 PSG results in patients with chronic insomnia. Our analysis revealed that participants with poorer rest-activity patterns (low IS and high IV) on actigraphy assessment exhibited higher insomnia severity scores compared to subjects with better rest-activity patterns (high IS and low IV). In addition, late-phase participants (late L5 and M10 onset times) exhibited higher insomnia severity scores, longer sleep latency, longer REM latency, and lower AHI compared to early-phase participants. Notably, these associations remained significant even after adjusting for covariates. We highlight the clinical implications and the possible adjunctive role of actigraphy-derived rest-activity patterns and circadian phase parameters for the performance and interpretation of PSG in patients with insomnia. Future studies using a larger sample size and repeated assessments of actigraphy and PSG are warranted.

## Methods

### Participants

This retrospective cross-sectional study used data from the database of a single university-affiliated hospital located in Seoul, South Korea, from May 2016 to January 2020. All patients in the database underwent overnight PSG and monitoring of actigraphy for at least 7 days from the night of PSG. In total, 124 participants diagnosed with chronic insomnia based on clinical symptoms and criteria of the Diagnostic and Statistical Manual of Mental Disorders (DSM-IV-TR) and International Classification of Sleep Disorders, Third Edition (ICSD-2) were consecutively selected. Inclusion criteria were individuals aged over 20 years with subjective sleep complaints. Exclusion criteria were as follows: non-Korean-speaking foreigners; shift workers; abnormal sleep–wake disorders; other sleep disorders, such as restless legs syndrome, narcolepsy, REM sleep behavior disorder, or periodic limb movement disorder, severe medical, neurological (neurodegenerative diseases, epilepsy, head injury), or psychiatric diseases (psychosis, current intake of antidepressants for diagnosed depression), and alcohol or illicit drug abuse or current intake of psychoactive medications. A total of 49 participants were excluded due to insufficient actigraphy data (less than 4 days, n = 32), shift worker or irregular sleep–wake rhythm (n = 13), and insomnia due to restless leg syndrome with or without definite periodic limb movement disorder (n = 4). Since we intended to assess the possible associations of actigraphy-derived rest-activity patterns and circadian phase parameters with PSG derived obstructive sleep apnea severity, we did not exclude participants with high AHI. Therefore, participants with combined insomnia with obstructive sleep apnea were included in our analysis. A final total of 75 participants were enrolled in this study.

### Standard protocol approval, registration, and patient consent

This study was approved by the Institutional Review Board of Samsung Medical Center (SMC IRB No. 2021-04-021). The need for patient informed consent was waived by Institutional Review Board of Samsung Medical Center due to the retrospective nature of the study. This study was performed in accordance with relevant guidelines and regulations of the IRB.

### Measurement of rest-activity patterns and circadian phase using actigraphy

Participants were instructed to wear a research-grade triaxial accelerometer Actiwatch 2 (Phillips Respironics, Murrysville, PA, USA) on their non-dominant wrist for at least 7 days prior to the night of PSG while performing their usual daily activities in a home setting. Activity counts in 1-min epochs from the first 7 consecutive days of data, commencing at midnight, were processed to calculate rest-activity patterns and circadian phase variables.

**Figure 3:**
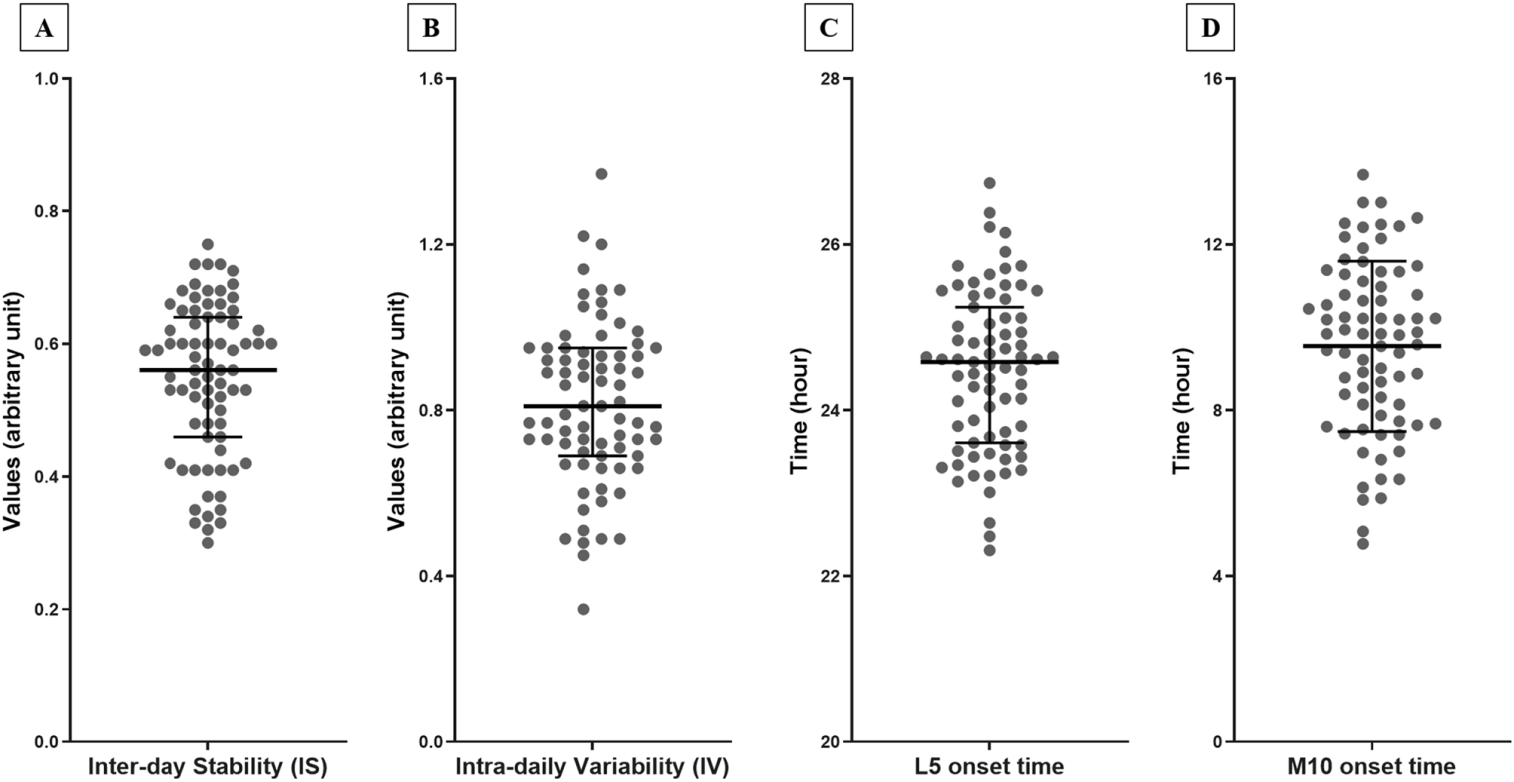
Distributions of rest-activity patterns and circadian phase variables. Rest-activity pattern variables are expressed as arbitrary units. Circadian phase variables are expressed as time units. Circles indicate patients. Bars in the middle indicate medians, while error bars indicate interquartile range. *IS* inter-daily stability, *IV* intra-daily variability, *L5 onset time* least active 5-h onset time, *M10 onset time* most active 10-h onset time.

Rest-activity patterns and circadian phase parameters were extracted using nonparametric analysis of actigraphy data. Nonparametric analysis does not adopt a priori assumptions about the waveform of daily activity; rather, it calculates variables based on raw activity counts^38^. The following parameters were calculated from the non-parametric analysis: (1) IS, which generally represents the strength of coupling of a rhythm to environmental zeitgebers; (2) IV, which generally represents activity fragmentation in a day; (3) L5 onset time; and (4) M10 onset time. Actigraphy data was handled using Actiware version 5.7 software and ‘nparACT’ package for R Statistical Software^39,40^. The distributions and correlations of four rest-activity patterns and circadian phase variables are presented in Fig. 3.

### Polysomnography

PSG measurements were recorded during one night of observation with standard electrodes and sensors using Embla N7000 (Medcare Flaga, Iceland). The following PSG parameters were measured and collected: TST, sleep latency, REM latency, WASO, percent time in each sleep stage (N1, N2, N3, and REM stages), and AHI. Participants completed the ISI and ESS questionnaires to measure the severity of subjectively reported insomnia symptoms and daytime sleepiness, respectively^41,42^.

### Statistical analysis

Continuous variable data were reported as mean and standard deviation (SD) or median and IQR after verifying the normality of the data distribution using the Shapiro–Wilk test. To explore possible associations between rest-activity patterns, circadian phase, PSG parameters, and clinical symptom scores, Pearson correlation analysis was performed for two normally distributed continuous variables. If one or both variables did not have a normal distribution, nonparametric Spearman correlation analysis was performed. Hierarchical clustering analyses were performed using Ward’s linkage algorithm with Euclidean distances for the actigraphy data-driven group classification of participants. For group comparisons, the Student’s t-test or Mann–Whitney U test was used for variables exhibiting a normal or non-normal distribution, respectively. ANCOVA was performed to examine the significance of observed associations after adjusting for potential covariates including age, sex, BMI, and lights-off time. Considering the skewed distribution and clinical implications of sleep latency, REM latency, and AHI on PSG, logistic regression analysis was performed using conventional cut-off scores after adjusting for age, sex, BMI, and lights-off time.
